# Improvement of genomic prediction by integrating additional single nucleotide polymorphisms selected from imputed whole genome sequencing data

**DOI:** 10.1038/s41437-019-0246-7

**Published:** 2019-07-05

**Authors:** Aoxing Liu, Mogens Sandø Lund, Didier Boichard, Emre Karaman, Sebastien Fritz, Gert Pedersen Aamand, Ulrik Sander Nielsen, Yachun Wang, Guosheng Su

**Affiliations:** 1 Department of Molecular Biology and Genetics, Aarhus University, 8830 Tjele, Denmark; 2 Key Laboratory of Animal Genetics, Breeding and Reproduction, MARA; National Engineering Laboratory for Animal Breeding, College of Animal Science and Technology, China Agricultural University, 100193 Beijing, PR China; 3 GABI, INRA, AGROParisTech, Université Paris Saclay, 78350 Jouy-en-Josas, France; 4 Nordic Cattle Genetic Evaluation, 8200 Aarhus N, Denmark; 5 Seges, 8200 Aarhus N, Denmark

**Keywords:** Animal breeding, Quantitative trait, Genetic markers

## Abstract

The availability of whole genome sequencing (WGS) data enables the discovery of causative single nucleotide polymorphisms (SNPs) or SNPs in high linkage disequilibrium with causative SNPs. This study investigated effects of integrating SNPs selected from imputed WGS data into the data of 54K chip on genomic prediction in Danish Jersey. The WGS SNPs, mainly including peaks of quantitative trait loci, structure variants, regulatory regions of genes, and SNPs within genes with strong effects predicted with variant effect predictor, were selected in previous analyses for dairy breeds in Denmark–Finland–Sweden (DFS) and France (FRA). Animals genotyped with 54K chip, standard LD chip, and customized LD chip which covered selected WGS SNPs and SNPs in the standard LD chip, were imputed to 54K together with DFS and FRA SNPs. Genomic best linear unbiased prediction (GBLUP) and Bayesian four-distribution mixture models considering 54K and selected WGS SNPs as one (a one-component model) or two separate genetic components (a two-component model) were used to predict breeding values. For milk production traits and mastitis, both DFS (0.025) and FRA (0.029) sets of additional WGS SNPs improved reliabilities, and inclusions of all selected WGS SNPs generally achieved highest improvements of reliabilities (0.034). A Bayesian four-distribution model yielded higher reliabilities than a GBLUP model for milk and protein, but extra gains in reliabilities from using selected WGS SNPs were smaller for a Bayesian four-distribution model than a GBLUP model. Generally, no significant difference was observed between one-component and two-component models, except for using GBLUP models for milk.

## Introduction

Genomic prediction has been widely applied in dairy cattle breeding (Hayes et al. 2009). To achieve reliable prediction for breeding values of candidate animals, a reference population consisted of a large number of individuals with both phenotypes and genotypes is required (Karaman et al. 2016). Assembling such a sufficiently large reference population, however, may not be possible for traits that are hard to measure, such as feed intake (Berry et al. 2014), or for breeds that are numerically small, such as Danish Jersey (Lund et al. 2016).

To improve reliabilities of genomic prediction, especially for a numerically small breed, many approaches have been investigated (Brøndum et al. 2015; Lund et al. 2016; van den Berg et al. 2016b). One approach is to increase SNP density in order to construct stronger linkage disequilibrium between single nucleotide polymorphisms (SNPs) and causative mutations (de Roos et al. 2008). However, reliabilities from using the HD chip were only a little higher than those from using the 54K chip (Su et al. 2012a; VanRaden et al. 2013), while reliabilities from using imputed whole genome sequencing (WGS) data were quite similar to those from using the HD chip (van Binsbergen et al. 2015). One possible reason could be that only causative mutations or SNPs very close to causative mutations can improve reliabilities of genomic prediction (van den Berg et al. 2016a). Moreover, the inclusion of a large number of noncausative SNPs may bring only noise to genomic prediction (Pérez-Enciso et al. 2015).

Instead of using all WGS SNPs, an alternative approach is to integrate only causative mutations or SNPs in high linkage disequilibrium with causative mutations into the genotype data of the 54K chip in genomic prediction (Brøndum et al. 2015; van den Berg et al. 2016a). Although most causative mutations in cattle remain unknown, large numbers of causative SNPs or SNPs in high linkage disequilibrium with causative SNPs have been detected from WGS data via quantitative trait loci (QTL) mapping (Daetwyler et al. 2014; Mao et al. 2016) and bioinformatics analyses (Michot et al. 2016; Boussaha et al. 2015). Benefits of incorporating QTL SNPs selected from WGS data in genomic prediction have been widely verified (Brøndum et al. 2015; van den Berg et al. 2016a), but less has been done for WGS SNPs selected from bioinformatics analyses. A customized LD chip was designed under the project of EuroGenomics, covering SNPs in the standard LD chip (Boichard et al. 2012) and more importantly, some additional WGS SNPs selected from peaks of QTL or bioinformatics analyses (Boichard et al. 2018). The selected WGS SNPs in this customized LD chip could be promising in genomic prediction but has not yet been investigated previously.

Sharing the reference population with other populations is another cost-effective approach to improve reliabilities, especially for a numerically small breed with limited bulls to be used (Lund et al. 2011). The magnitudes of improvements on reliabilities depend on how well the information of causative mutations could be captured by using a joint reference population (Lund et al. 2014). A simulation study showed that directly using selected WGS SNPs close to causative mutations improved reliability in genomic prediction with a joint reference population, but reliability dropped quickly when using the WGS SNPs distant from causative mutations (van den Berg et al. 2016a). In real data, however, the option of using selected WGS SNPs has not yet been investigated in genomic prediction with a joint reference population. Another strategy that can increase the reference population size is to include cows in the reference. Although the phenotypic information of cows is less precise (more noise) than those of bulls with a large number of progenies, improvements of reliabilities can still be achieved since a large number of cows are available to be genotyped and included in the reference (Buch et al. 2012; Su et al. 2016). With the increase of reference population size, however, the improvement of reliabilities from using additional information from other sources (e.g., selected WGS SNPs) could be reduced (Daetwyler et al. 2008).

Different models in terms of different assumptions for SNP effects could influence the efficiency of using selected WGS SNPs in genomic prediction (Brøndum et al. 2015). The model with assumption closest to the true distribution of SNP effects can achieve the highest reliability. The Genomic best linear unbiased prediction (GBLUP) model implicitly assumes that SNP effects follow a normal distribution with a null mean and an equal variance, and therefore, a same amount of shrinkage is implicitly applied to all SNPs. The Bayesian mixture model such as Bayesian four-distribution mixture model (Erbe et al. 2012; Gao et al. 2013), however, assumes a prior distribution that SNP effects follow a mixture of four normal distributions with null means but varying variance parameters, resulting in different shrinkages for SNPs in different classes. Reliabilities from Bayesian mixture models are expected to be higher than GBLUP models for traits with major QTL, but similar to GBLUP models for traits affected by many QTL each with small effect. Furthermore, selected WGS SNPs can be considered as a genetic component together with standard chip SNPs (a one-component model) or as a separate genetic component (a two-component model) (Visscher et al. 2007; Brøndum et al. 2015). A two-component model will likely be more beneficial in a GBLUP model than in a Bayesian mixture model (Brøndum et al. 2015), since different variances between standard chip SNPs and selected WGS SNPs have already been allowed in a Bayesian mixture model but not in a GBLUP model.

Primary objectives of this study were to investigate the effects of selected WGS SNPs on genomic prediction in Danish Jersey. Besides, we investigated the effects of using selected WGS SNPs along with the increase of the reference population size by including US Jersey bulls and Danish Jersey cows. Furthermore, we assessed different models on their efficiency to use the information of selected WGS SNPs.

## Materials and methods

### Data

#### Genotype

A total of 3745 Danish Jersey bulls, 1168 US Jersey bulls, and 28,678 Danish Jersey cows were genotyped. The Danish Jersey bulls were mainly genotyped with the Illumina Bovine SNP50 chip (54K, Illumina, Inc). The US Jersey bulls were genotyped with either the Illumina Bovine SNP50 chip or the GeneSeek Genomic Profiler HD chip (777K, GeneSeek, Neogen Corporation), but SNPs which were not in the Bovine SNP50 chip were excluded in this study. For Danish Jersey cows, 3% were genotyped with Illumina Bovine SNP50 chip, 49% with standard Bovine LD Chip (standard LD, Illumina, Inc.), and 48% with EuroGenomics customized Illumina Bovine LD chip (Boichard et al. 2018). The EuroGenomics customized LD chip included SNPs in the standard LD chip together with 1754 WGS SNPs selected by Denmark–Finland–Sweden (DFS) and 4325 WGS SNPs selected by France (FRA). The DFS SNPs were peaks of QTL detected from imputed WGS data in Nordic Holsteins, Nordic Red, and Danish Jersey, selecting SNPs within each breed according to *P*-values of a single-marker regression model, functional annotations and linkage disequilibrium between SNPs (Brøndum et al. 2015). The FRA SNPs were WGS SNPs selected from one of the following categories: (i) literature; (ii) within genes with strong effect predicted with variant effect predictor (McLaren et al. 2016) (e.g., frameshift, stop gain, splicing site, and nonsynonymous substitution with strong predicted effect); (iii) regulatory regions of genes; (iv) peaks of QTL; and (v) breakpoints of structural SNPs (Boichard et al. 2018). Both DFS and FRA SNPs were discovered for milk production and functional traits (e.g., fertility, mastitis, calving, growth, and longevity). The SNPs on the sex chromosome or with unknown positions, monomorphism, multiple alleles and minor allele frequency (MAF) lower than 0.01 were excluded.

Animals genotyped with 54K and different versions of LD chips were imputed to 54K + DFS + FRA by a two-step family and population-based approach using the FImpute software (Sargolzaei et al. 2014). Firstly, individuals genotyped with different versions of LD chips were imputed to 54K. Secondly, individuals with 54K or imputed 54K were imputed to 54K + DFS + FRA. The SNP-wise imputation accuracy was measured as the Pearson correlation between observed and imputed genotypes (coded as 0, 1, or 2), and the proportion of correctly imputed genotypes to all imputed genotypes (i.e., concordance rate). Only SNPs with both correlation and concordance rate higher than 0.8 were used in genomic prediction. Ultimately, 39,803 SNPs in the 54K chip, 1270 DFS SNPs and 2427 FRA SNPs were kept for genomic prediction, with 28 SNPs overlapped between DFS and FRA SNPs. The correlations of imputation for SNPs used in genomic prediction were 97.0% for standard LD chip to 54K, 96.9% for DFS SNPs and 95.9% for FRA SNPs; while concordance rates were 98.3% for standard LD chip to 54K, 98.1% for DFS SNPs and 97.8% for FRA SNPs.

#### Phenotype

The analyzed traits included milk, protein, fat, mastitis, and fertility. Deregressed proof (DRP) derived from official EBV was used as the pseudo phenotype in genomic prediction. To avoid double counting of the information from the derivation of DRP and the prediction of breeding values, two sets of DRP were used in this study. One derived from all genotyped Danish and US Jersey bulls (DRP_b_) to be used for genomic prediction with bulls as the reference population and as the validation population; the other derived from all genotyped Danish and US Jersey bulls and Danish Jersey cows (DRP_bc_) to be used for genomic prediction with the reference populations including cows and cows as the validation population. Thus, DRP of bulls in DRP_b_ set could include the information of the genotyped cows, while those in DRP_bc_ set not. Reliability of DRP was calculated as $r_{\rm{DRP}}^2 = \frac{{ERC_i}}{{ERC_i + \lambda }}$, where $\lambda = \frac{{1 - h^2}}{{h^2}}$. The *ERC*_*i*_ was the effective record contribution of *i*th animal and *h*^2^ was heritability which was 0.390 for milk, fat, and protein, 0.066 for mastitis, and 0.064 for fertility.

### Statistical models for predicting breeding values

A linear mixed model using pedigree (PBLUP) or genome-wide marker (GBLUP) based relationships and a Bayesian four-distribution mixture model were used for predicting breeding values. In GBLUP and Bayesian four-distribution mixture model, effects of selected WGS SNPs on genomic prediction were investigated by comparing four SNP scenarios: (i) 54K; (ii) 54K + DFS; (iii) 54K + FRA; and (iv) 54K + DFS + FRA. Furthermore, we assessed a one-component model and a two-component model on their efficiency to use the information of selected WGS SNPs. A one-component model considering all SNPs as one genetic component was applied for all four SNP scenarios; while a two-component model considering 54K SNPs and selected WGS SNPs as two separate genetic components was applied only for scenarios including selected WGS SNPs. It was assumed that SNPs captured all genetic variations, and therefore, the residual polygenic effect was not included in the models.

#### PBLUP model

The PBLUP model is

1 $$\bf {\mathbf{y}} = 1\mu + {\mathbf{Za}} + {\mathbf{e}},$$

where **y** is the vector of DRPs; **1** is the vector of ones; *μ* is the overall mean; **a** is the vector of additive genetic effects; **Z** is the incidence matrix relating **a** to phenotypes; and **e** is the vector of random residuals. It was assumed that ${\mathbf{a}}\sim N\left( {0,{\mathbf{A}}{\mathrm{\sigma }}_{\mathrm{a}}^2} \right)$ and ${\mathbf{e}}\sim {\boldsymbol{N}}\left( {0,{\mathbf{D}}{\mathrm{\sigma }}_e^2} \right)$. The **A** is the additive relationship matrix constructed from the pedigree which traced genotyped animals three generations back. The **D** is the diagonal matrix with elements $d_{jj} = \left( {1 - r_{\mathrm{DRP}}^2} \right){\mathrm{/}}r_{\mathrm{DRP}}^2$ to account for heterogeneous residual variances (${\mathrm{\sigma }}_e^2$) due to different reliabilities of DRP ($r_{\mathrm{DRP}}^2$). The estimation of variance components, and the prediction of breeding values with PBLUP models were performed using the DMU software (Madsen and Jensen 2012).

#### GBLUP model

The one-component GBLUP (G1) model is

2 $$\bf {\mathbf{y}} = 1\mu + {\mathbf{Za}} + {\mathbf{e}}.$$

The two-component GBLUP (G2) model is

3 $$\bf {\mathbf{y}} = 1\mu + {\mathbf{Za}}_{54{\mathbf{K}}} + {\mathbf{Za}}_{{\mathbf{WGS}}} + {\mathbf{e}},$$

where **y** is the vector of DRPs; **1** is the vector of ones; *μ* is the overall mean; **a**, **a**_**54K**_, and **a**_**WGS**_ are vectors of additive genetic effects accounted by all SNPs in the model, by 54K SNPs and by selected WGS SNPs; **Z** is the incidence matrix relating **a**, **a**_**54K**_, and **a**_**WGS**_ to phenotypes; and **e** is the vector of random residuals. It is assumed that ${\mathbf{a}}\sim {\boldsymbol{N}}\left( {0,{\mathbf{G}}{\mathrm{\sigma }}_{\mathrm{a}}^2} \right)$, ${\mathbf{a}}_{54{\mathbf{K}}}\sim {\boldsymbol{N}}( {0,{\mathbf{G}}_{54{\mathbf{K}}}{\mathrm{\sigma }}_{{\mathrm{a}}_{54{\mathrm{K}}}}^2})$, ${\mathbf{a}}_{{\mathbf{WGS}}}\sim {\boldsymbol{N}}( {0,{\mathbf{G}}_{{\mathbf{WGS}}}{\mathrm{\sigma }}_{{\mathrm{a}}_{{\mathrm{WGS}}}}^2})$, and ${\mathbf{e}}\sim {\boldsymbol{N}}\left( {0,{\mathbf{D}}{\mathrm{\sigma }}_e^2} \right)$. The **G**, **G**_**54K**_, and **G**_**WGS**_ are genomic relationship matrices constructed from all SNPs in the model, from 54K SNPs and from selected WGS SNPs using method 1 in VanRaden (2008); ${\mathrm{\sigma }}_{\mathrm{a}}^2$, ${\mathrm{\sigma }}_{{\mathrm{a}}_{54{\mathrm{K}}}}^2$ and ${\mathrm{\sigma }}_{{\mathrm{a}}_{{\mathrm{WGS}}}}^2$ are additive genetic variances explained by SNPs in **G**, **G**_**54K**_, and **G**_**WGS**_, respectively. The **D** is the diagonal matrix with elements $d_{jj} = \left( {1 - r_{\mathrm{DRP}}^2} \right)/r_{\mathrm{DRP}}^2$ to account for heterogeneous residual variances (${\mathrm{\sigma }}_e^2$) due to different reliabilities of DRP ($r_{\mathrm{DRP}}^2$). In the G2 model, proportions of total variances explained by 54K SNPs and selected WGS SNPs are estimated from the data, while the covariance between 54K SNPs and selected WGS SNPs is assumed to be zero. The estimation of variance components, and the prediction of breeding values with GBLUP models were performed using the DMU software (Madsen and Jensen 2012).

#### Bayesian four-distribution mixture model

The one-component Bayesian four-distribution mixture (B1) model is

4 $${\boldsymbol{y}} = 1\mu + {\mathbf{Xg}} + {\boldsymbol{e}}.$$

The two-component Bayesian four-distribution mixture (B2) model is

5 $${\boldsymbol{y}} = 1\mu + {\mathbf{X}}_{54{\mathbf{K}}}{\mathbf{g}}_{54{\mathbf{K}}} + {\mathbf{X}}_{{\mathbf{WGS}}}{\mathbf{g}}_{{\mathbf{WGS}}} + {\mathbf{e}},$$

where **y** is the vector of DRPs; **1** is the vector of ones; *μ* is the overall mean; **g**, **g**_**54K**_, and **g**_**WGS**_ are vectors of effects for all SNPs in the model, 54K SNPs, and selected WGS SNPs;**X**, **X**_**54K**_, and **X**_**WGS**_ are genotype matrices for all SNPs in the model, 54K SNPs, and selected WGS SNPs; and **e** is the vector of random residuals. It is assumed that the distribution of marker effects (**g**, **g**_**54K**_, or **g**_**QTL**_) follows a mixture of four normal distributions:

$${\mathbf{g}}_{\mathbf{i}}\sim \pi _{i1}N\left( {0,{\mathbf{I}}\sigma _{i1}^2} \right) + \pi _{i2}N\left( {0,{\mathbf{I}}\sigma _{i2}^2} \right) + \pi _{i3}N\left( {0,{\mathbf{I}}\sigma _{i3}^2} \right) + \pi _{i4}N\left( {0,{\mathbf{I}}\sigma _{i4}^2} \right),$$

where *i* is the *i*th genetic component in the model; *π*_*ij*_ (*j* = 1,2,3 and 4) is the probability of an SNP belongs to the *j*th distribution within the *i*th component, and $\sigma _{ij}^2$ is the variance for *j*th distribution within the *i*th component. In the present study, *π*_*ij*_ is sampled from the Dirichlet distribution *π*_*ij*_ = (*π*_*i*1_, *π*_*i*2_, *π*_*i*3_, *π*_*i*4_) ~ dir (125, 25, 5, 1) with prior *π*_*i*1_ = 0.889, *π*_*i*2_ = 0.1, *π*_*i*3_ = 0.01, and *π*_*i*4_ = 0.001, where $\sigma _{ij}^2$ is updated from the data with $1000\sigma _{i1}^2 = 100\sigma _{i2}^2 = 10\sigma _{i3}^2 = \sigma _{i4}^2$. The ratios among $\sigma _{i1}^2$, $\sigma _{i2}^2$, $\sigma _{i3}^2$, and $\sigma _{i4}^2$ are fixed, thus, only one of them is required to be estimated within each genetic component. It is assumed that ${\mathbf{e}}\sim {\boldsymbol{N}}\left( {0,{\mathbf{D}}{\mathrm{\sigma }}_e^2} \right)$, where **D** is the diagonal matrix with elements $d_{jj} = \left( {1 - r_{\mathrm{DRP}}^2} \right)/r_{\mathrm{DRP}}^2$ to account for heterogeneous residual variances (${\mathrm{\sigma }}_e^2$) due to different reliabilities of DRP ($r_{\rm{DRP}}^2$). In the B2 model, proportions of total variances explained by 54K SNPs and selected WGS SNPs are estimated from the data, while the covariance between 54K SNPs and selected WGS SNPs is assumed to be zero. Each of the Bayesian four-distribution mixture model was run as a single chain with a total length of 50,000 Markov chain samples, where the first 10,000 iterations were discarded as burn-in. Ultimately, every 20th sample of the remaining 40,000 iterations were saved for the posterior analysis. The analyses with Bayesian four-distribution mixture models were performed using the Bayz software (http://www.bayz.biz).

### Validation of genomic prediction

#### Reference and validation populations

To investigate the effects of using selected WGS SNPs on genomic prediction along with the change of the reference population size by including US Jersey bulls and Danish Jersey cows, five reference populations were tested in this study: (i) Danish bulls (DK); (ii) Danish and US bulls (DKUS); (iii) Danish cows (COW); (iv) Danish bulls and cows (DKCOW); and (v) Danish and US bulls and Danish cows (DKUSCOW). It is improper to include bulls in the validation population given cows in the reference, since most genotyped cows were daughters or sibs for genotyped bulls. Therefore, cows were used as the validation population. To avoid strong sib-relationships between reference and validation populations, a strategy similar to that in Su et al. (2016) was used to create validation and reference populations. Genotyped cows born in and after 2014 and their genotyped paternal female half-sibs born after July 1st 2008 were considered as the validation cows. Half-sib families with size larger than 500 were removed from the validation set and kept in the reference set to avoid a large reduction of the reference population size. Finally, the validation population included 5829 validation cows from 155 paternal half-sib families. For the reference populations, validation cows’ maternal female and male half-sibs born after July 1st 2008 and progenies of validation cows and the sibs of these progenies were excluded. For fertility, however, DRP was only available in bulls. The validation population for fertility was 281 Danish bulls born in and after 2005, while reference populations were: (i) 1029 Danish bulls born before 2005 (DK), and (ii) a combination of 1029 Danish bulls and 1153 US bulls (DKUS). Only animals with the reliability of DRP higher than 0.20 were used as the validation population. Validation and reference scenarios as well as numbers of animals in validation and reference populations for all traits are presented in Table 1. Reliabilities of DRP in reference and validation populations are presented in Table 2.

**Table 1:** Number of animals in reference and validation populations

Traits	Validation population	Reference population			
		Scenarios	Danish bulls	US bulls	Danish cows
Milk, protein, fat, and mastitis	5829 Danish cows	DK	1282	–	–
		DKUS	1282	1148	–
		COW	–	–	8763
		DKCOW	1282	–	8602
		DKUSCOW	1282	1148	8602
Fertility	281 Danish bulls	DK	1029	–	–
		DKUS	1029	1157	–

*DK* Danish bulls as the reference population, *DKUS* Danish and US bulls as the reference population, *COW* Danish cows as the reference population, *DKCOW* Danish bulls and cow as the reference population, *DKUSCOW* Danish and US bulls and Danish cows as the reference population

**Table 2:** Reliability of DRP ($r_{\rm{DRP}}^2$) in reference and validation populations

Trait	*h*^2^	$r_{\rm{DRP}}^2$					
		DK	DKUS	COW	DKCOW	DKUSCOW	Validation population
Milk	0.390	0.921	0.846	0.487	0.543	0.565	0.446
Protein	0.390	0.921	0.846	0.487	0.543	0.565	0.446
Fat	0.390	0.921	0.846	0.487	0.543	0.565	0.446
Mastitis	0.066	0.806	0.738	0.233	0.307	0.341	0.240
Fertility^a^	0.064	0.624	0.609	–	–	–	0.661

*DK* Danish bulls as the reference population, *DKUS* Danish and US bulls as the reference population, *COW* Danish cows as the reference population, *DKCOW* Danish bulls and cow as the reference population, *DKUSCOW* Danish and US bulls and Danish cows as the reference population

^a^Bulls were used as the validation population

#### Reliability, bias, and stability

The predictability for estimating breeding values was assessed by reliability, bias, and stability. The reliability of prediction was measured as the squared correlation between estimated breeding values and DRP divided by the average reliability of DRP for the animals in the validation population. The bias of prediction was measured as the regression coefficient of DRP on the estimated breeding values for the animals in the validation population. The stability of prediction was measured as the correlation between breeding values estimated from the reduced dataset (using DRP from the reference population) and the full dataset (using DRP from both reference and validation populations) for the animals in the validation population, which was performed in scenarios of 54K and 54K + DFS + FRA using GBLUP models.

We compared reliability (or bias) among different SNP sets given the same reference population and model, among different reference populations given the same SNP set and model, and among different models given the same reference population and SNP set. A nonparametric bootstrap sample, with an equal size as the validation population, was obtained by randomly sampling with replacement from validation animals. We repeated the bootstrap procedure to get 10,000 bootstrap samples. The standard deviation of reliability (or bias) and the contrasts from 10,000 bootstrap samples were used as the standard error of reliability (or bias) and the contrasts. A two-tailed paired *t*-test was used to compare reliability (or bias) between a pair of scenarios. A Bonferroni correction was used to control the false positive caused by multiple comparisons.

### Comparison of genomic prediction with or without the selected WGS SNPs while keeping the same number of SNPs

The SNP density increased by adding additional selected WGS SNPs. For example, the number of SNPs used for genomic prediction increased 3669 by adding DFS + FRA SNPs. We hypothesized that changes in reliabilities (or bias) after adding selected WGS SNPs was due to these SNPs being or linking closely to causative mutations instead of the increase in SNP density. To test this hypothesis, we randomly removed 3669 SNPs from 54K and created a new SNP scenario together with DFS and FRA SNPs (54Kminus + DFS + FRA). Thus, the number of SNPs in 54Kminus+DFS+FRA was the same as that in 54K. We repeated this procedure five times. The average reliability from five replicates of 54Kminus+DFS+FRA for each model and each reference population was compared with the reliability from the 54K using the same model and the same reference population.

## Results

Reliabilities of predicting breeding values using a PBLUP model and a G1 model are presented in Table 3. Using relationships derived from SNPs yielded much higher reliabilities (0.388 average across all SNP sets) compared with using relationships derived from pedigree (0.173) for all traits. For PBLUP, standard errors of reliabilities for mastitis (~1.5 times more) and fertility (~2.5 times more) were much larger than those for milk production traits, indicating that the estimates of reliabilities for mastitis and fertility were less precise than those for milk production traits. Compared with using 54K SNPs alone, adding additional selected WGS SNPs led to significant improvements of reliabilities for milk and protein, small improvements for fat and mastitis, whereas no improvement for fertility. For milk production and mastitis, both DFS and FRA sets of additional WGS SNPs improved reliability (an average gain of 0.025 and 0.029, respectively), and the inclusion of all selected WGS SNPs generally achieved the highest reliabilities (an average gain of 0.034). Generally, reliabilities improved along with the increase of the reference population size when using the same SNP set. Including US Jersey bulls in the reference (DKUS) led to significant improvements (0.068 over all SNP sets) of reliabilities compared with only using a DK reference for milk, protein, and fat. Including cows in the reference population led to significant improvements in reliabilities compared with only using bulls as reference. By expanding the reference population from the DK reference to the DKCOW reference, the average improvement of reliability across all traits over all SNP sets was 0.148, and from the DKUS reference to the DKUSCOW reference was 0.109. Furthermore, for milk production and mastitis, improvements of reliabilities by integrating selected WGS SNPs ranged from 0.023 to 0.037 when using different reference populations. The gains in order were DKUSCOW<COW<DKCOW<DK<DKUS.

**Table 3:** Reliabilities from a PBLUP^a^ model and a G1^b^ model using different SNP scenarios^c^ with significance tests^d^

Trait	Reference	PBLUP	G1			
			54K	54K + DFS	54K + FRA	54K + DFS + FRA
Milk	DK	0.132 (0.013)	^e^0.320_d_	^e^0.397_c_	^e^0.403_b_	^e^0.424_a_
	DKUS	0.174 (0.015)	^d^0.426_d_	^d^0.510_c_	^d^0.519_b_	^d^0.533_a_
	COW	0.105 (0.012)	^c^0.577_d_	^c^0.637_c_	^c^0.645_b_	^c^0.649_a_
	DKCOW	0.149 (0.014)	^b^0.619_c_	^b^0.679_b_	^b^0.684_b_	^b^0.691_a_
	DKUSCOW	0.189 (0.016)	^a^0.655_d_	^a^0.704_c_	^a^0.710_b_	^a^0.715_a_
Protein	DK	0.175 (0.015)	^d^0.268_d_	^d^0.291_c_	^d^0.298_b_	^d^0.306_a_
	DKUS	0.209 (0.016)	^c^0.326_d_	^c^0.351_c_	^c^0.361_b_	^c^0.368_a_
	COW	0.116 (0.013)	^c^0.366_c_	^c^0.389_b_	^c^0.397_a_	^c^0.399_a_
	DKCOW	0.150 (0.014)	^b^0.402_c_	^b^0.427_b_	^b^0.432_b_	^b^0.435_a_
	DKUSCOW	0.178 (0.015)	^a^0.429_c_	^a^0.449_b_	^a^0.454_ab_	^a^0.456_a_
Fat	DK	0.199 (0.016)	^c^0.265_c_	^c^0.277_ab_	^c^0.276_b_	^c^0.281_a_
	DKUS	0.214 (0.016)	^b^0.296_c_	^b^0.308_b_	^b^0.313_b_	^b^0.316_a_
	COW	0.148 (0.014)	^b^0.333_c_	^b^0.340_b_	^b^0.341_ab_	^b^0.343_a_
	DKCOW	0.222 (0.017)	^a^0.376_a_	^a^0.380_a_	^a^0.381_a_	^a^0.382_a_
	DKUSCOW	0.231 (0.017)	^a^0.384_a_	^a^0.389_a_	^a^0.389_a_	^a^0.391_a_
Mastitis	DK	0.205 (0.035)	^c^0.254_a_	^c^0.256_a_	^b^0.256_a_	^b^0.258_a_
	DKUS	0.196 (0.034)	^bc^0.267_a_	^bc^0.268_a_	^b^0.268_a_	^b^0.269_a_
	COW	0.168 (0.031)	^abc^0.295_a_	^abc^0.297_a_	^ab^0.298_a_	^ab^0.299_a_
	DKCOW	0.154 (0.030)	^ab^0.321_b_	^ab^0.323_b_	^a^0.331_a_	^a^0.332_a_
	DKUSCOW	0.146 (0.030)	^a^0.323_b_	^a^0.323_b_	^a^0.333_a_	^a^0.333_a_
Fertility^e^	DK	0.161 (0.050)	^a^0.301_a_	^a^0.298_a_	^a^0.291_a_	^a^0.288_a_
	DKUS	0.189 (0.053)	^a^0.303_a_	^a^0.302_a_	^a^0.294_a_	^a^0.293_a_

*DK* Danish bull as the reference population, *DKUS* Danish and US bull as the reference population, *COW* Danish cows as the reference population, *DKCOW* Danish bull and cows as the reference population, *DKUSCOW* Danish and US bulls and Danish cows as the reference population

^a^PBLUP: pedigree BLUP

^b^G1: one-component GBLUP

^c^54K: SNPs in 54K chip; 54K+DFS: SNPs in 54K chip together with WGS SNPs selected by analysis of data from major dairy breeds in Denmark–Finland–Sweden; 54K + FRA: SNPs in 54K chip together with WGS SNPs selected by analysis of data from major dairy breeds in France; 54K + DFS + FRA: SNPs in 54K chip together with WGS SNPs selected by analysis of data from major dairy breeds in Denmark–Finland–Sweden and France

^d^Letters in the superscript were for comparisons among reference populations using the same SNP scenario and model; letters in subscript were for comparisons among SNP scenarios using the same reference populations and model. Same letters denote no significant difference; while different letters denote significant difference at *P* = 0.05 after Bonferroni correction

^e^Bulls were used as the validation population

Reliabilities in genomic prediction from different models for 54K and 54K + DFS + FRA are presented in Table 4. When using 54K, the B1 model was significantly superior to a G1 model for milk, protein, and fat, but equal to a G1 model for mastitis and fertility. In this case, the average improvement of reliability from a G1 model to a B1 model for milk, protein, and fat was 0.051. When using 54K +DFS + FRA, a B1 model was significantly superior to a G1 model for milk and protein but equal to a G1 model for fat, mastitis, and fertility. In this case, the average improvement of reliability from a G1 model to a B1 model for milk and protein was 0.023. Although a B1 model was better than a G1 model for milk and protein, the extra gain in reliability from using selected WGS SNPs (DFS+FRA) was smaller for a B1 model (0.011) than a G1 model (0.059). Regarding the comparisons between a one-component model and a two-component model when using 54K + DFS + FRA, significant differences were observed only for milk with DK, DKUS, and DKUSCOW as reference. For milk, a G2 model was generally superior to a G1 model (0.016), whereas a B2 model was equal to a B1 model.

**Table 4:** Reliabilities for 54K^a^ and 54K + DFS + FRA^b^ using different models^c^, with significance tests^d^

Trait	Reference	54K		54K+DFS+FRA			
		G1	B1	G1	B1	G2	B2
Milk	DK	0.320_b_	0.463_a_	0.424_c_	0.484_a_	0.463_b_	0.489_a_
	DKUS	0.426_b_	0.549_a_	0.533_c_	0.576_a_	0.552_b_	0.586_a_
	COW	0.577_b_	0.667_a_	0.649_b_	0.674_a_	0.652_b_	0.675_a_
	DKCOW	0.619_b_	0.704_a_	0.691_c_	0.721_a_	0.703_b_	0.723_a_
	DKUSCOW	0.655_b_	0.732_a_	0.715_b_	0.741_a_	0.722_b_	0.742_a_
Protein	DK	0.268_b_	0.299_a_	0.306_b_	0.314_a_	0.309_ab_	0.305_ab_
	DKUS	0.326_b_	0.378_a_	0.368_b_	0.383_a_	0.371_b_	0.375_ab_
	COW	0.366_b_	0.396_a_	0.399_a_	0.400_a_	0.394_a_	0.401_a_
	DKCOW	0.402_b_	0.440_a_	0.435_b_	0.444_a_	0.439_ab_	0.446_a_
	DKUSCOW	0.429_b_	0.465_a_	0.456_b_	0.468_a_	0.457_b_	0.468_a_
Fat	DK	0.265_b_	0.278_a_	0.281_a_	0.284_a_	0.276_a_	0.277_a_
	DKUS	0.296_b_	0.314_a_	0.316_ab_	0.318_a_	0.311_ab_	0.312_b_
	COW	0.333_b_	0.343_a_	0.343_a_	0.347_a_	0.345_a_	0.348_a_
	DKCOW	0.376_a_	0.382_a_	0.382_a_	0.384_a_	0.382_a_	0.384_a_
	DKUSCOW	0.384_b_	0.398_a_	0.391_ab_	0.398_a_	0.391_b_	0.397_ab_
Mastitis	DK	0.254_a_	0.252_a_	0.258_a_	0.243_ab_	0.250_a_	0.226_b_
	DKUS	0.267_a_	0.267_a_	0.269_a_	0.262_a_	0.261_ab_	0.245_b_
	COW	0.295_a_	0.296_a_	0.299_a_	0.298_a_	0.296_a_	0.296_a_
	DKCOW	0.321_a_	0.323_a_	0.332_a_	0.328_a_	0.329_a_	0.327_a_
	DKUSCOW	0.323_a_	0.328_a_	0.333_a_	0.334_a_	0.332_a_	0.337_a_
Fertility^e^	DK	0.301_a_	0.292_a_	0.288_ab_	0.276_a_	0.267_ab_	0.243_b_
	DKUS	0.303_a_	0.300_a_	0.293_a_	0.292_a_	0.288_a_	0.281_a_

*DK* Danish bull as the reference population, *DKUS* Danish and US bull as the reference population, *COW* Danish cows as the reference population, *DKCOW* Danish bull and cows as the reference population. *DKUSCOW* Danish and US bulls and Danish cows as the reference population

^a^54K: SNPs in the 54K chip

^b^54K + DFS + FRA: SNPs in 54K chip together with WGS SNPs selected by analysis of data from major dairy breeds in Denmark–Finland–Sweden and France

^c^G1: one-component GBLUP model; G2: two-component GBLUP model; B1: one-component Bayesian four-distribution mixture model; B2: two-component Bayesian four-distribution mixture model

^d^Letters in the right lower position were for comparisons among models using the same reference population and SNP scenario. Same letters denote no significant difference; while different letters denote significant difference

^e^Bulls were used as the validation population

Variance components estimated from a G1 model and a G2 model are presented in additional Table 1. In the G1 model, differences between variances components estimated before and after adding selected WGS SNPs were small. In the G2 model, proportions of genetic variances explained by selected WGS SNPs were 40.2% for milk, 31.2% for protein, 23.6% for fat, 17.5% for mastitis, and 25.5% for fertility, average on all scenarios with different reference populations and SNP sets. Total additive genetic variances (the sum of two genetic components) from the G2 model were in general slightly smaller than those from the G1 model, suggesting the covariance between the two genetic components could not be zero. The variances estimated from different sets of phenotypic data were somewhat different due to different structures in different data sets.

Bias for predicting breeding values using a PBLUP model and a G1 model is presented in Table 5. When cows were included in the reference population, regression coefficients were lower from GBLUP models than from PBLUP models. When only bulls were in the reference population, no clear trend in the regression coefficient was observed between GBLUP and PBLUP models. Using selected WGS SNPs generally led to equal or significantly increased regression coefficients compared with using only 54K SNPs. Regression coefficients further deviated from unity indicated more bias. Regression coefficients increased from a DK to a DKUS reference and from a DKCOW to a DKUSCOW reference for all traits, except for from a DKCOW to a DKUSCOW reference for mastitis. Besides, the inclusion of cows in the reference led to equal or significantly decreased regression coefficients when using genomic information. Bias of genomic prediction from different models for 54K and 54K + DFS + FRA is presented in Table 6. Generally, there was no clear trend for bias observed from different models.

**Table 5:** Regression coefficients of DRP on prediction from a PBLUP^a^ model and a G1^b^ model using different SNP scenarios^c^ with significance tests^d^

Trait	Reference	PBLUP	G1			
			54K	54K + DFS	54K + FRA	54K + DFS + FRA
Milk	DK	1.03 (0.05)	^ab^1.07_c_	^a^1.16_b_	^a^1.17_b_	^a^1.18_a_
	DKUS	1.12 (0.05)	^a^1.12_c_	^a^1.19_b_	^a^1.20_ab_	^a^1.21_a_
	COW	1.10 (0.06)	^c^0.94_a_	^d^0.92_b_	^d^0.92_ab_	^d^0.92_ab_
	DKCOW	1.12 (0.05)	^b^1.05_a_	^c^1.04_a_	^c^1.04_a_	^c^1.04_a_
	DKUSCOW	1.21 (0.05)	^a^1.07_a_	^b^1.07_a_	^b^1.07_a_	^b^1.07_a_
Protein	DK	1.00 (0.04)	^cd^0.87_c_	^cd^0.90_b_	^cd^0.91_ab_	^bc^0.91_a_
	DKUS	1.12 (0.04)	^ab^0.93_c_	^ab^0.96_b_	^ab^0.97_ab_	^a^0.98_a_
	COW	1.09 (0.06)	^abcd^0.95_a_	^abcd^0.93_a_	^abcd^0.94_a_	^abc^0.94_a_
	DKCOW	0.93 (0.04)	^bd^0.91_a_	^bd^0.92_a_	^bd^0.92_a_	^c^0.92_a_
	DKUSCOW	1.02 (0.04)	^ac^0.93_a_	^ac^0.94_a_	^ac^0.94_a_	^ab^0.94_a_
Fat	DK	0.96 (0.04)	^bd^0.81_a_	^ab^0.82_a_	^bd^0.82_a_	^ab^0.82_a_
	DKUS	1.06 (0.04)	^ac^0.85_a_	^ab^0.84_a_	^ac^0.85_a_	^ab^0.85_a_
	COW	0.94 (0.05)	^ab^0.85_a_	^a^0.86_a_	^ab^0.86_a_	^a^0.86_a_
	DKCOW	1.00 (0.04)	^cd^0.82_a_	^b^0.82_a_	^cd^0.82_a_	^b^0.82_a_
	DKUSCOW	1.04 (0.04)	^abcd^0.83_a_	^ab^0.83_a_	^abcd^0.83_a_	^ab^0.83_a_
Mastitis	DK	1.37 (0.12)	^ab^1.12_a_	^a^1.12_a_	^a^1.12_a_	^a^1.12_a_
	DKUS	1.39 (0.12)	^a^1.17_a_	^a^1.17_a_	^a^1.16_a_	^a^1.16_a_
	COW	1.46 (0.14)	^bc^1.00_a_	^ab^1.00_a_	^abc^1.00_a_	^abc^1.00_a_
	DKCOW	1.21 (0.12)	^cd^0.95_a_	^b^0.95_a_	^b^0.96_a_	^b^0.96_a_
	DKUSCOW	1.07 (0.12)	^d^0.90_a_	^c^0.90_a_	^c^0.91_a_	^c^0.91_a_
Fertility^e^	DK	0.90 (0.15)	^a^1.10_a_	^a^1.09_a_	^a^1.08_a_	^a^1.07_a_
	DKUS	0.92 (0.14)	^a^1.05_a_	^a^1.05_a_	^a^1.02_a_	^a^1.03_a_

*DK* Danish bull as the reference population, *DKUS* Danish and US bull as the reference population, *COW* Danish cows as the reference population, *DKCOW* Danish bull and cows as the reference population, *DKUSCOW* Danish and US bulls and Danish cows as the reference population

^a^PBLUP: pedigree BLUP

^b^G1: one-component GBLUP

^c^54K: SNPs in 54K chip; 54K + DFS: SNPs in 54K chip together with WGS SNPs selected by analysis of data from major dairy breeds in Denmark–Finland–Sweden; 54K + FRA: SNPs in 54K chip together with WGS SNPs selected by analysis of data from major dairy breeds in France; 54K +DFS + FRA: SNPs in 54K chip together with WGS SNPs selected by analysis of data from major dairy breeds in Denmark–Finland–Sweden and France

^d^Letters in the left upper positions were for comparisons among reference populations using the same SNP scenario and model; letters in the right lower position were for comparisons among SNP scenarios using the same reference populations and model. Same letters denote no significant difference, while different letters denote significant difference

^e^Bulls were used as the validation population

**Table 6:** Regression coefficients of DRP on prediction from 54K^a^ and 54K + DFS + FRA^b^ using different models^c^, with significance tests^d^

Trait	Reference	54K		54K +DFS + FRA			
		G1	B1	G1	B1	G2	B2
Milk	DK	1.07_b_	1.20_a_	1.18_ab_	1.23_a_	1.16_c_	1.20_b_
	DKUS	1.12_b_	1.19_a_	1.21_bc_	1.22_ab_	1.20_c_	1.23_a_
	COW	0.94_a_	0.91_b_	0.92_a_	0.91_a_	0.91_a_	0.91_a_
	DKCOW	1.05_a_	1.04_a_	1.04_a_	1.04_a_	1.04_a_	1.04_a_
	DKUSCOW	1.07_a_	1.08_a_	1.07_a_	1.08_a_	1.07_a_	1.08_a_
Protein	DK	0.87_b_	0.92_a_	0.91_ab_	0.92_a_	0.89_bc_	0.87_c_
	DKUS	0.93_b_	0.98_a_	0.98_ab_	0.98_a_	0.97_ab_	0.96_b_
	COW	0.95_a_	0.92_b_	0.94_a_	0.91_b_	0.92_b_	0.91_b_
	DKCOW	0.91_a_	0.92_a_	0.92_a_	0.92_a_	0.92_a_	0.92_a_
	DKUSCOW	0.93_b_	0.95_a_	0.94_a_	0.95_a_	0.94_a_	0.95_a_
Fat	DK	0.81_a_	0.82_a_	0.82_a_	0.81_a_	0.79_b_	0.79_b_
	DKUS	0.85_a_	0.83_a_	0.85_a_	0.84_ab_	0.83_bc_	0.82_c_
	COW	0.85_a_	0.85_a_	0.86_a_	0.86_a_	0.87_a_	0.86_a_
	DKCOW	0.82_a_	0.82_a_	0.82_a_	0.82_a_	0.82_a_	0.82_a_
	DKUSCOW	0.83_a_	0.84_a_	0.83_a_	0.84_a_	0.83_a_	0.84_a_
Mastitis	DK	1.12_a_	1.12_a_	1.12_a_	1.09_a_	1.09_a_	1.01_b_
	DKUS	1.17_a_	1.15_a_	1.16_a_	1.13a_b_	1.13_b_	1.07_c_
	COW	1.00_a_	1.00_a_	1.00_a_	1.00_a_	1.00_a_	1.00_a_
	DKCOW	0.95_a_	0.94_a_	0.96_a_	0.95_a_	0.96_a_	0.95_a_
	DKUSCOW	0.90_b_	0.93_a_	0.91_b_	0.94_a_	0.91_b_	0.95_a_
Fertility^e^	DK	1.10_a_	1.07_a_	1.07_a_	1.03_ab_	1.02_b_	0.92_c_
	DKUS	1.05_a_	1.04_a_	1.03_ab_	1.03_a_	1.01_ab_	1.00_b_

*DK* Danish bull as the reference population, *DKUS* Danish and US bull as the reference population, *COW* Danish cows as the reference population, *DKCOW* Danish bull and cows as the reference population. DKUSCOW Danish and US bulls and Danish cows as the reference population

^a^54K: SNPs in the 54K chip

^b^54K + DFS + FRA: SNPs in 54K chip together with WGS SNPs selected by analysis of data from major dairy breeds in Denmark–Finland–Sweden and France

^c^G1: one-component GBLUP model; G2: two-component GBLUP model; B1: one-component Bayesian four-distribution mixture model; B2: two-component Bayesian four-distribution mixture model

^d^Letters in the right lower position were for comparisons among models using the same reference population and SNP scenario. Same letters denote no significant difference; while different letters denote significant difference

^e^Bulls were used as the validation population

Stabilities of predicting breeding values for 54K and 54K + DFS + FRA using GBLUP models are presented in additional Table 2. The average correlation between predictions from full and reduced datasets was 0.834 using 54K SNPs with G1 models, while it was 0.851 and 0.856 using 54K + DFS + FRA SNPs with G1 and G2 models, respectively. In general, adding selected WGS SNPs improved prediction stabilities. The G2 model yielded better stabilities than the G1 model for milk, fat, and protein but not for mastitis. For scenarios using different reference populations, stabilities increased with the increase of the reference population size, ranging from 0.764 for using a DK reference to 0.909 for using a DKUSCOW reference.

Differences between 54Kminus+DFS+FRA and 54K in reliabilities and bias using G1 and B1 models are presented in additional Tables 3 and 4. Generally, differences in reliabilities (or bias) between 54Kminus+DFS+FRA and 54K were similar to those between 54K + DFS + FRA and 54K. Improvements in reliabilities from 54K to 54Kminus+DFS+FRA were 0.082 for milk, 0.034 for protein, 0.012 for fat, and 0.006 for mastitis using G1 models, while those were 0.016 for milk, 0.006 for protein, 0.002 for fat, and no improvement for mastitis using B1 models. There was no improvement for fertility using both G1 and B1 models. Differences in bias between 54Kminus+DFS+FRA and 54K were no more than 0.05 using both G1 and B1 models except for milk from DK (0.10) and DKUS reference (0.10) using G1 models.

## Discussion

In this study, we investigated the effects of integrating selected WGS SNPs in the EuroGenomics customized LD chip (Boichard et al. 2018) to 54K SNPs on genomic prediction in Danish Jersey, which is a numerically small breed. Using selected WGS SNPs improved reliabilities and stabilities for milk production and mastitis, which was consistent with the results of integrating QTL SNPs in genomic prediction for Nordic Holsteins and Red (Brøndum et al. 2015). Besides, differences between 54Kminus+DFS+FRA and 54K were similar to those between 54K + DFS + FRA and 54K, supporting the hypothesis that improvements of reliabilities from integrating selected WGS SNPs were due to selected WGS SNPs being or linking closely to causative mutations, not due to the increase of SNP density. For fertility, however, using selected WGS SNPs did not improve the reliability, which was in line with the previous study in Nordic Holsteins and Red (Brøndum et al. 2015) where the improvement of reliability for fertility was limited when integrating selected WGS SNPs. Due to the polygenic nature of fertility traits (Liu et al. 2017) and the small reference population size, the power of detecting causative mutations from WGS data could be limited in Danish Jersey. Moreover, more WGS SNPs were selected for milk production traits (about two times more) than for fertility in DFS SNPs, since the number of WGS SNPs to be selected was determined by the economic weight of a specific trait in Nordic selection index (Brøndum et al. 2015).

To select a model with high efficiency to use the information of selected WGS SNPs, we compared a GBLUP model with a Bayesian four-distribution mixture model, and treating the selected WGS SNPs and 54K SNPs as the same or different genetic components. The B1 model used in this study was similar to the BayesR model which has been verified to be equal or superior to a G1 model in various species (Erbe et al. 2012). Improvements of reliabilities from selected WGS SNPs, however, were smaller for B1 models compared with G1 models since B1 models can better identify SNPs linking closely to causative mutations than G1 models when using 54K SNPs. Furthermore, for milk, a G2 model was generally superior to a G1 model which was in line with Brøndum et al. (2015), but no significant difference was observed between reliabilities from B1 and B2 models. The Bayesian mixture models already allowed to account for different variances between SNPs in the 54K chip and selected WGS SNPs when using a B1 model, and thus a B2 model worked similarly to a B1 model. The idea of putting more emphases on informative SNPs by a two-component model was similar to a weighted GBLUP model (Zhang et al. 2010), which has been verified to be better than the regular GBLUP model (Su et al. 2014). The weighted GBLUP model has similar computation costs as the regular GBLUP model but is able to reach similar reliabilities as the Bayesian mixture model, therefore, could be easily implemented in the industry. In addition, integrating DFS and FRA SNPs in a single-step model with weights on the genomic relationship matrix could further improve reliabilities for both genotyped and nongenotyped animals. A simulation study showed that a weighted single-step model efficiently used the information of causative SNPs when weighted by realistic effects or estimated effects from association studies (Fragomeni et al. 2017). Furthermore, selected WGS SNPs used in this study were discovered based on different strategies, e.g., QTL detection and bioinformatics analysis. The model which can incorporate prior biological information by defining classes of SNPs, e.g., BayesRS (Brøndum et al. 2012) and BayesRC (MacLeod et al. 2016), is likely to better use the information of causative mutations and further improve the reliability.

To test whether the benefits of integrating selected WGS SNPs still existed when increasing the reference population size, we applied multiple reference populations in genomic prediction. Reliabilities for prediction improved after including US Jersey in the reference for all traits. A joint reference with genetically related populations led to improvements in reliabilities since linkage disequilibrium persisted over long distances. For example, large improvements of reliabilities were observed when pooling Danish Jersey with US Jersey (Wiggans et al. 2015; Su et al. 2016), whereas limited or no improvement of reliability was observed when pooling Jersey with Holsteins (Lund et al. 2014). To date, the information of US Jersey bulls has been included in the routine genomic evaluation for Danish Jersey. Improvements of reliabilities for milk production traits from integrating selected WGS SNPs were almost the same when the reference population increased from DK to DKUS. The most pronounced advantage of using WGS data is in genomic prediction across populations (Iheshiulor et al. 2016), and it has been reported that selected WGS SNPs can improve genomic prediction across populations (van den Berg et al. 2016a).

Including cows in the reference population led to significant improvements of reliabilities compared with only using bulls as reference for all traits, which was in line with results from previous studies using both real (Su et al. 2016) and simulated data (Buch et al. 2012). Although the phenotypic information of cows is less informative compared with those of bulls with a large number of progenies, improvements of reliabilities were still considerable since a large number of genotyped cows were added to the reference population. In this study, reliabilities of using COW as a reference population were higher than those from using DKUS as a reference population, this could be due to that genotyped Jersey cows were much less selected than genotyped Jersey bulls. It has been reported that selective animals could underestimate reliabilities of genomic prediction (Uimari and Mäntysaari 1993; Su et al. 2012b). Improvements of reliabilities for milk production traits from using selected WGS SNPs slightly dropped after including cows in the reference. It has been reported that improvements of reliabilities from using additional information from other sources depended on the size of the original reference population (Daetwyler et al. 2008). Therefore, improvements of reliabilities from using selected WGS SNPs were dropped given the reference population dramatically enlarged by including a large number of cows. Furthermore, even in the largest reference population (i.e., DKUSCOW), benefits of using selected WGS SNPs for milk and protein remained significant. This indicated that selected WGS SNPs could also benefit for populations or breeds with relatively large population sizes.

An alternative strategy to use information from other populations is to preselect informative WGS SNPs from the population with large population size or from multiple breeds (van den Berg et al. 2016b), because of large power to detect informative SNPs. Besides, FRA SNPs performed slightly better than DFS SNPs although with poorer imputation accuracy. However, we cannot conclude that SNPs detected from bioinformatics analyses (e.g., FRA SNPs) were more meaningful in genomic prediction than those from QTL mapping (e.g., DFS SNPs) since the number of FRA SNPs was around two times of DFS SNPs. A combination of DFS and FRA SNPs yielded the highest reliabilities, which indicated that more reliable predictions as well as faster genetic gains could be achieved if the information of selected WGS SNPs can be shared across countries. In addition, other novel strategies, e.g., machine learning (Long et al. 2007), could be promising for the preselection of WGS SNPs for genomic prediction.

A total of 117 DFS SNPs and 568 FRA SNPs with MAF higher than 0.01 were removed from analyses due to low imputation accuracy. Although selected WGS SNPs used in genomic prediction (after quality control) had relatively high imputation accuracy, the imputation accuracy was still much lower than unity. Compared with true genotypes, imputed genotypes decreased reliabilities and unbiasedness of genomic prediction, where the degree of decrement was influenced by the imputation accuracy (van den Berg et al. 2017). Improvement of reliability in genomic prediction from using selected WGS SNPs could be enlarged if more animals were directly genotyped with customized LD chip instead of imputation.

In summary, based on the results of this study, the efforts for developing and implementing the customized SNP chip with WGS SNPs selected from QTL mapping and/or bioinformatics are worthwhile in the industry, especially for the numerically small breed. Firstly, improvements in reliabilities from integrating selected WGS SNPs are promising in general. Secondly, the inclusion of thousands of additional WGS SNPs would not lead to a large increase of the computational burden, and therefore easy to implement in the industry. Thirdly, benefiting from the development of genotyping technologies, the costs of the customized SNP chip (e.g., customized LD chip) is similar to that of the standard SNP chip (e.g., standard LD chip), which makes the improvements of reliabilities from integrating selected WGS SNPs being an additional bonus. Since genetic progress by selection is linearly related to accuracy of genetic evaluation, considering a large dairy cattle population, even a small improvement in reliability is important for breeding and production.

## Conclusion

Integrating additional selected WGS SNPs to the genotype data of 54K chip led to significant improvements of reliabilities for milk and protein, small improvements for fat and mastitis, and no improvement for fertility. Reliabilities improved along with the increase of the reference population size for all traits, and benefits from using selected WGS SNPs for milk and protein remained significant in the scenario of the largest reference population. A Bayesian four-distribution model yielded higher reliabilities than a GBLUP model for milk and protein, but extra gains in reliabilities from using selected WGS SNPs was smaller for a Bayesian four-distribution model than a GBLUP model. No significant difference was observed between considering 54K SNPs and selected WGS SNPs as one or two genetic components, except for using GBLUP models for milk.

## Supplementary information

Additional tables
