# Supplementary material for: Improvement of genomic prediction by integrating additional single nucleotide polymorphisms selected from imputed whole genome sequencing data
Source: Heredity (Edinb). 2019 Jul 5;124(1):37–49. doi: 10.1038/s41437-019-0246-7 (PMC6906477; doi:10.1038/s41437-019-0246-7)
Supplement: Supplementary file 1 — Additional tables [file 41437_2019_246_MOESM1_ESM.docx]

*Additional Table 1. Variance components (*V_G1,_ V_G2_, V_E_*)^1^ for de-regressed proof using different SNP sets^2^ estimated from G1 and G2 models^3^.*

| Trait | Reference^4^ | SNP | Model | V_G1_ | V_G2_ |  | V_E_ |
| --- | --- | --- | --- | --- | --- | --- | --- |
| Milk | DK | 54K | G1 | 74.6 (5.4) | -- |  | 10.3 (1.6) |
|  | DK | 54K+DFS | G1 | 74.8 (5.2) | -- |  | 8.6 (1.5) |
|  | DK | 54K+FRA | G1 | 75.2 (5.3) | -- |  | 8.8 (1.5) |
|  | DK | 54K+DFS+FRA | G1 | 74.2 (5.1) | -- |  | 8.3 (1.4) |
|  | DK | 54K+DFS+FRA | G2 | 31.0 (4.9) | 37.5 (5.1) |  | 10.2 (1.4) |
|  | DKUS | 54K | G1 | 79.2 (4.1) | -- |  | 12.9 (0.9) |
|  | DKUS | 54K+DFS | G1 | 75.9 (3.8) | -- |  | 11.7 (0.8) |
|  | DKUS | 54K+FRA | G1 | 77.2 (3.9) | -- |  | 11.6 (0.8) |
|  | DKUS | 54K+DFS+FRA | G1 | 74.7 (3.8) | -- |  | 11.6 (0.8) |
|  | DKUS | 54K+DFS+FRA | G2 | 43.9 (3.7) | 26.8 (3.5) |  | 12.5 (0.8) |
|  | COW | 54K | G1 | 127.3 (5.9) | -- |  | 101.0 (2.1) |
|  | COW | 54K+DFS | G1 | 114.5 (5.3) | -- |  | 96.2 (2.0) |
|  | COW | 54K+FRA | G1 | 114.7 (5.3) | -- |  | 95.9 (1.9) |
|  | COW | 54K+DFS+FRA | G1 | 109.3 (5.1) | -- |  | 96.0 (1.9) |
|  | COW | 54K+DFS+FRA | G2 | 54.2 (4.2) | 40.0 (3.9) |  | 99.3 (2.0) |
|  | DKCOW | 54K | G1 | 111.0 (4.4) | -- |  | 38.5 (0.7) |
|  | DKCOW | 54K+DFS | G1 | 101.0 (4.0) | -- |  | 37.0 (0.7) |
|  | DKCOW | 54K+FRA | G1 | 101.6 (4.0) | -- |  | 36.9 (0.7) |
|  | DKCOW | 54K+DFS+FRA | G1 | 97.6 (3.9) | -- |  | 36.9 (0.7) |
|  | DKCOW | 54K+DFS+FRA | G2 | 59.3 (3.4) | 32.1 (3.1) |  | 37.6 (0.7) |
|  | DKUSCOW | 54K | G1 | 106.9 (3.9) | -- |  | 38.2 (0.7) |
|  | DKUSCOW | 54K+DFS | G1 | 94.5 (3.5) | -- |  | 37.0 (0.7) |
|  | DKUSCOW | 54K+FRA | G1 | 95.4 (3.5) | -- |  | 36.8 (0.7) |
|  | DKUSCOW | 54K+DFS+FRA | G1 | 91.7 (3.4) | -- |  | 36.9 (0.7) |
|  | DKUSCOW | 54K+DFS+FRA | G2 | 60.1 (3.1) | 26.6 (2.6) |  | 37.5 (0.7) |
| Protein | DK | 54K | G1 | 95.8 (6.7) | -- |  | 11.8 (1.9) |
|  | DK | 54K+DFS | G1 | 95.7 (6.6) | -- |  | 10.9 (1.8) |
|  | DK | 54K+FRA | G1 | 94.7 (6.6) | -- |  | 11.0 (1.8) |
|  | DK | 54K+DFS+FRA | G1 | 94.1 (6.5) | -- |  | 10.7 (1.8) |
|  | DK | 54K+DFS+FRA | G2 | 49.4 (6.6) | 41.0 (6.4) |  | 12.3 (1.8) |
|  | DKUS | 54K | G1 | 94.3 (5.0) | -- |  | 17.3 (1.1) |
|  | DKUS | 54K+DFS | G1 | 92.6 (4.9) | -- |  | 16.8 (1.1) |
|  | DKUS | 54K+FRA | G1 | 92.5 (4.8) | -- |  | 16.7 (1.1) |
|  | DKUS | 54K+DFS+FRA | G1 | 90.9 (4.8) | -- |  | 16.7 (1.1) |
|  | DKUS | 54K+DFS+FRA | G2 | 60.9 (5.0) | 27.7 (4.3) |  | 17.5 (1.1) |
|  | COW | 54K | G1 | 76.6 (4.4) | -- |  | 120.0 (2.3) |
|  | COW | 54K+DFS | G1 | 75.9 (4.3) | -- |  | 117.4 (2.2) |
|  | COW | 54K+FRA | G1 | 74.7 (4.2) | -- |  | 117.3 (2.2) |
|  | COW | 54K+DFS+FRA | G1 | 73.3 (4.2) | -- |  | 117.3 (2.2) |
|  | COW | 54K+DFS+FRA | G2 | 44.4 (3.9) | 22.7 (3.1) |  | 119.1 (2.2) |
|  | DKCOW | 54K | G1 | 98.9 (4.0) | -- |  | 44.0 (0.8) |
|  | DKCOW | 54K+DFS | G1 | 96.1 (3.9) | -- |  | 43.2 (0.8) |
|  | DKCOW | 54K+FRA | G1 | 95.4 (3.9) | -- |  | 43.3 (0.8) |
|  | DKCOW | 54K+DFS+FRA | G1 | 93.7 (3.8) | -- |  | 43.3 (0.8) |
|  | DKCOW | 54K+DFS+FRA | G2 | 69.0 (3.8) | 22.5 (2.9) |  | 43.7 (0.8) |
|  | DKUSCOW | 54K | G1 | 98.9 (3.8) | -- |  | 44.2 (0.8) |
|  | DKUSCOW | 54K+DFS | G1 | 95.3 (3.6) | -- |  | 43.7 (0.8) |
|  | DKUSCOW | 54K+FRA | G1 | 95.0 (3.6) | -- |  | 43.6 (0.8) |
|  | DKUSCOW | 54K+DFS+FRA | G1 | 93.2 (3.6) | -- |  | 43.6 (0.8) |
|  | DKUSCOW | 54K+DFS+FRA | G2 | 72.6 (3.6) | 19.0 (2.5) |  | 44.0 (0.8) |
| Fat | DK | 54K | G1 | 112.9 (7.7) | -- |  | 12.7 (2.1) |
|  | DK | 54K+DFS | G1 | 110.5 (7.4) | -- |  | 12.3 (2.0) |
|  | DK | 54K+FRA | G1 | 111.2 (7.5) | -- |  | 12.3 (2.0) |
|  | DK | 54K+DFS+FRA | G1 | 109.6 (7.4) | -- |  | 12.2 (2.0) |
|  | DK | 54K+DFS+FRA | G2 | 67.4 (8.0) | 39.1 (7.1) |  | 13.7 (2.0) |
|  | DKUS | 54K | G1 | 110.3 (5.8) | -- |  | 19.3 (1.3) |
|  | DKUS | 54K+DFS | G1 | 107.5 (5.6) | -- |  | 18.3 (1.2) |
|  | DKUS | 54K+FRA | G1 | 108.5 (5.6) | -- |  | 18.1 (1.2) |
|  | DKUS | 54K+DFS+FRA | G1 | 106.3 (5.5) | -- |  | 18.2 (1.2) |
|  | DKUS | 54K+DFS+FRA | G2 | 74.8 (5.8) | 29.0 (4.8) |  | 19.0 (1.2) |
|  | COW | 54K | G1 | 83.3 (5.0) | -- |  | 145.4 (2.8) |
|  | COW | 54K+DFS | G1 | 81.6 (4.9) | -- |  | 145.3 (2.7) |
|  | COW | 54K+FRA | G1 | 81.6 (5.0) | -- |  | 145.3 (2.7) |
|  | COW | 54K+DFS+FRA | G1 | 80.8 (4.9) | -- |  | 145.4 (2.7) |
|  | COW | 54K+DFS+FRA | G2 | 65.4 (5.2) | 13.9 (3.2) |  | 146.1 (2.8) |
|  | DKCOW | 54K | G1 | 114.7 (4.8) | -- |  | 53.9 (1.0) |
|  | DKCOW | 54K+DFS | G1 | 110.7 (4.6) | -- |  | 54.0 (1.0) |
|  | DKCOW | 54K+FRA | G1 | 111.6 (4.7) | -- |  | 53.9 (1.0) |
|  | DKCOW | 54K+DFS+FRA | G1 | 110.0 (4.6) | -- |  | 54.0 (1.0) |
|  | DKCOW | 54K+DFS+FRA | G2 | 88.9 (4.8) | 20.1 (3.4) |  | 54.3 (1.0) |
|  | DKUSCOW | 54K | G1 | 114.4 (4.4) | -- |  | 53.5 (0.9) |
|  | DKUSCOW | 54K+DFS | G1 | 108.8 (4.2) | -- |  | 53.6 (0.9) |
|  | DKUSCOW | 54K+FRA | G1 | 109.9 (4.3) | -- |  | 53.4 (0.9) |
|  | DKUSCOW | 54K+DFS+FRA | G1 | 107.9 (4.2) | -- |  | 53.6 (0.9) |
|  | DKUSCOW | 54K+DFS+FRA | G2 | 88.4 (4.3) | 18.6 (2.9) |  | 53.9 (0.9) |
| Mastitis | DK | 54K | G1 | 63.9 (6.4) | -- |  | 26.5 (2.2) |
|  | DK | 54K+DFS | G1 | 63.6 (6.4) | -- |  | 26.4 (2.2) |
|  | DK | 54K+FRA | G1 | 63.9 (6.4) | -- |  | 26.5 (2.2) |
|  | DK | 54K+DFS+FRA | G1 | 63.7 (6.4) | -- |  | 26.4 (2.2) |
|  | DK | 54K+DFS+FRA | G2 | 41.8 (7.7) | 20.6 (6.5) |  | 27.1 (2.2) |
|  | DKUS | 54K | G1 | 60.3 (5.4) | -- |  | 51.8 (2.3) |
|  | DKUS | 54K+DFS | G1 | 60.2 (5.3) | -- |  | 51.6 (2.3) |
|  | DKUS | 54K+FRA | G1 | 60.5 (5.3) | -- |  | 51.5 (2.3) |
|  | DKUS | 54K+DFS+FRA | G1 | 60.4 (5.3) | -- |  | 51.4 (2.3) |
|  | DKUS | 54K+DFS+FRA | G2 | 41.1 (6.2) | 18.5 (5.1) |  | 52.0 (2.3) |
|  | COW | 54K | G1 | 82.2 (7.2) | -- |  | 347.5 (6.2) |
|  | COW | 54K+DFS | G1 | 82.1 (7.2) | -- |  | 347.5 (6.2) |
|  | COW | 54K+FRA | G1 | 82.9 (7.2) | -- |  | 347.3 (6.2) |
|  | COW | 54K+DFS+FRA | G1 | 82.8 (7.2) | -- |  | 347.4 (6.2) |
|  | COW | 54K+DFS+FRA | G2 | 81.6 (8.2) | 0.65 (4.2) |  | 347.5 (6.2) |
|  | DKCOW | 54K | G1 | 93.3 (5.2) | -- |  | 95.9 (1.6) |
|  | DKCOW | 54K+DFS | G1 | 92.8 (5.2) | -- |  | 95.9 (1.6) |
|  | DKCOW | 54K+FRA | G1 | 94.2 (5.2) | -- |  | 95.7 (1.6) |
|  | DKCOW | 54K+DFS+FRA | G1 | 93.7 (5.2) | -- |  | 95.7 (1.6) |
|  | DKCOW | 54K+DFS+FRA | G2 | 83.2 (5.9) | 10.3 (3.8) |  | 95.8 (1.6) |
|  | DKUSCOW | 54K | G1 | 109.1 (5.4) | -- |  | 94.7 (1.6) |
|  | DKUSCOW | 54K+DFS | G1 | 108.6 (5.3) | -- |  | 94.7 (1.6) |
|  | DKUSCOW | 54K+FRA | G1 | 109.5 (5.4) | -- |  | 94.5 (1.6) |
|  | DKUSCOW | 54K+DFS+FRA | G1 | 109.2 (5.4) | -- |  | 94.5 (1.6) |
|  | DKUSCOW | 54K+DFS+FRA | G2 | 96.5 (5.8) | 12.9 (3.7) |  | 94.6 (1.6) |
| Fertility^5^ | DK | 54K | G1 | 153.8 (19.7) | -- |  | 74.3 (5.9) |
|  | DK | 54K+DFS | G1 | 154.3 (19.7) | -- |  | 73.9 (5.9) |
|  | DK | 54K+FRA | G1 | 154.2 (19.7) | -- |  | 73.7 (5.9) |
|  | DK | 54K+DFS+FRA | G1 | 154.7 (19.7) | -- |  | 73.4 (5.9) |
|  | DK | 54K+DFS+FRA | G2 | 106.0 (24.9) | 48.8 (24.2) |  | 74.0 (5.8) |
|  | DKUS | 54K | G1 | 142.5 (13.8) | -- |  | 116.7 (5.5) |
|  | DKUS | 54K+DFS | G1 | 142.5 (13.8) | -- |  | 116.7 (5.5) |
|  | DKUS | 54K+FRA | G1 | 143.6 (13.8) | -- |  | 115.7 (5.5) |
|  | DKUS | 54K+DFS+FRA | G1 | 143.5 (13.8) | -- |  | 115.7 (5.5) |
|  | DKUS | 54K+DFS+FRA | G2 | 115.5 (16.0) | 27.8 (11.3) |  | 116.2 (5.5) |

^1^V_G1_ = additive genetic variance estimated from a G1 model or from a G2 model for the first genetic component (54K); V_G2_ = additive genetic variance estimated from a G2 model for the second genetic component (selected WGS SNPs); V_E_ = residual variance.

^2^54K: SNPs in 54K chip; 54K + DFS: SNPs in 54K chip together with WGS SNPs selected by analysis of data from major dairy breeds in Denmark-Finland-Sweden; 54K + FRA: SNPs in 54K chip together with WGS SNPs selected by analysis of data from major dairy breeds in Denmark-Finland-Sweden and France ; 54K + DFS + FRA: SNPs in 54K chip together with WGS SNPs selected by analysis of data from major dairy breeds in Denmark-Finland-Sweden and France.

^3^G1: one-component GBLUP model; G2: two-component GBLUP model.

^4^DK: Danish bull as the reference population; DKUS: Danish and US bull as the reference population; COW = Danish cows as the reference population; DKCOW = Danish bull and cows as the reference population. DKUSCOW= Danish and US bulls and Danish cows as the reference population.

^5^Bulls were used as the validation population.

*Attached Table 2. Correlations between predictions from full and reference data based on 54K^1^ and 54K + DFS + FRA^2^ using G1 and G2 models^3^.*

| Trait | Reference^4^ | 54K |  | 54K+ DFS +FRA | |  |  |
| --- | --- | --- | --- | --- | --- | --- | --- |
|  |  | G1 |  | G1 | G2 |  |  |
| Milk | DK | 0.715 |  | 0.762 | 0.800 |  |  |
|  | DKUS | 0.813 |  | 0.853 | 0.870 |  |  |
|  | COW | 0.860 |  | 0.891 | 0.902 |  |  |
|  | DKCOW | 0.903 |  | 0.927 | 0.935 |  |  |
|  | DKUSCOW | 0.925 |  | 0.941 | 0.946 |  |  |
| Protein | DK | 0.737 |  | 0.761 | 0.771 |  |  |
|  | DKUS | 0.810 |  | 0.831 | 0.837 |  |  |
|  | COW | 0.815 |  | 0.834 | 0.838 |  |  |
|  | DKCOW | 0.877 |  | 0.893 | 0.898 |  |  |
|  | DKUSCOW | 0.898 |  | 0.909 | 0.911 |  |  |
| Fat | DK | 0.743 |  | 0.763 | 0.763 |  |  |
|  | DKUS | 0.795 |  | 0.818 | 0.820 |  |  |
|  | COW | 0.829 |  | 0.838 | 0.842 |  |  |
|  | DKCOW | 0.880 |  | 0.887 | 0.889 |  |  |
|  | DKUSCOW | 0.890 |  | 0.898 | 0.899 |  |  |
| Mastitis | DK | 0.784 |  | 0.789 | 0.783 |  |  |
|  | DKUS | 0.807 |  | 0.812 | 0.803 |  |  |
|  | COW | 0.817 |  | 0.819 | 0.815 |  |  |
|  | DKCOW | 0.886 |  | 0.891 | 0.889 |  |  |
|  | DKUSCOW | 0.896 |  | 0.900 | 0.899 |  |  |

^1^54K: SNPs in 54K chip.

^2^54K + DFS + FRA: SNPs in 54K chip together with WGS SNPs selected by analysis of data from major dairy breeds in Denmark-Finland-Sweden and France.

^3^G1: one-component GBLUP model; G2: two-component GBLUP model.

^4^DK: Danish bull as the reference population; DKUS: Danish and US bull as the reference population; COW = Danish cows as the reference population; DKCOW = Danish bull and cows as the reference population. DKUSCOW= Danish and US bulls and Danish cows as the reference population.

*Attached Table 3. Differences in reliabilities (A - B) between 54Kminus + DFS + FRA^1^ (A) and 54K^2^ (B) using G1 and B1 models^3^.*

| Trait | Reference^4^ | G1 | B1 |
| --- | --- | --- | --- |
| Milk | DK | 0.100 (0.002) | 0.018 (0.003) |
|  | DKUS | 0.105 (0.001) | 0.027 (0.001) |
|  | COW | 0.072 (0.000) | 0.008 (0.001) |
|  | DKCOW | 0.072 (0.000) | 0.019 (0.000) |
|  | DKUSCOW | 0.060 (0.000) | 0.009 (0.001) |
| Protein | DK | 0.036 (0.001) | 0.013 (0.001) |
|  | DKUS | 0.041 (0.001) | 0.005 (0.001) |
|  | COW | 0.033 (0.000) | 0.003 (0.001) |
|  | DKCOW | 0.032 (0.000) | 0.004 (0.000) |
|  | DKUSCOW | 0.027 (0.000) | 0.004 (0.001) |
| Fat | DK | 0.016 (0.000) | 0.006 (0.001) |
|  | DKUS | 0.020 (0.0001) | -0.004 (0.001) |
|  | COW | 0.010 (0.001) | 0.004 (0.001) |
|  | DKCOW | 0.006 (0.000) | 0.003 (0.001) |
|  | DKUSCOW | 0.007 (0.000) | 0.001 (0.001) |
| Mastitis | DK | 0.004 (0.001) | -0.010 (0.001) |
|  | DKUS | 0.002 (0.002) | -0.004 (0.002) |
|  | COW | 0.004 (0.001) | 0.003 (0.002) |
|  | DKCOW | 0.011 (0.001) | 0.006 (0.001) |
|  | DKUSCOW | 0.010 (0.002) | -0.006 (0.001) |
| Fertility^5^ | DK | -0.012 (0.003) | 0.003 (0.003) |
|  | DKUS | -0.010 (0.002) | -0.002 (0.002) |

^1^54Kminus + DFS + FRA: SNPs in 54K chip excluded 3,669 randomly removed SNPs, together with WGS SNPs selected by analysis of data from major dairy breeds in Denmark-Finland-Sweden and France.

^2^54K: SNPs in 54K chip.

^3^G1: one-component GBLUP model; B1: one-component Bayesian four-distribution mixture model.

^4^DK: Danish bull as the reference population; DKUS: Danish and US bull as the reference population; COW = Danish cows as the reference population; DKCOW = Danish bull and cows as the reference population. DKUSCOW= Danish and US bulls and Danish cows as the reference population.

^5^Bulls were used as the validation population.

*Attached Table 4. Differences in regression coefficients of de-regressed profs on predictions (A - B) between 54Kminus + DFS + FRA^1^ (A) and 54K^2^ (B) using G1 and B1 models^3^.*

| Trait | Reference^4^ | G1 | B1 |
| --- | --- | --- | --- |
| Milk | DK | 0.10 (0.00) | 0.03 (0.00) |
|  | DKUS | 0.09 (0.00) | 0.03 (0.00) |
|  | COW | -0.02 (0.00) | 0.00 (0.00) |
|  | DKCOW | -0.01 (0.00) | 0.00 (0.00) |
|  | DKUSCOW | 0.00 (0.00) | 0.00 (0.00) |
| Protein | DK | 0.03 (0.00) | 0.00 (0.00) |
|  | DKUS | 0.05 (0.00) | 0.00 (0.00) |
|  | COW | -0.01 (0.00) | -0.01 (0.00) |
|  | DKCOW | 0.01 (0.00) | 0.00 (0.00) |
|  | DKUSCOW | 0.01 (0.00) | 0.00 (0.00) |
| Fat | DK | 0.01 (0.00) | -0.02 (0.00) |
|  | DKUS | 0.00 (0.00) | 0.02 (0.01) |
|  | COW | -0.01 (0.00) | 0.01 (0.00) |
|  | DKCOW | 0.00 (0.00) | 0.00 (0.00) |
|  | DKUSCOW | 0.00 (0.00) | 0.00 (0.00) |
| Mastitis | DK | 0.00 (0.00) | -0.03 (0.00) |
|  | DKUS | -0.02 (0.01) | -0.02 (0.01) |
|  | COW | -0.01 (0.00) | -0.01 (0.01) |
|  | DKCOW | 0.01 (0.00) | 0.01 (0.00) |
|  | DKUSCOW | 0.01 (0.01) | 0.01 (0.00) |
| Fertility^5^ | DK | -0.04 (0.01) | -0.03 (0.01) |
|  | DKUS | -0.02 (0.01) | -0.01 (0.00) |

^1^54Kminus + DFS + FRA: SNPs in 54K chip excluded 3,669 randomly removed SNPs, together with WGS SNPs selected by analysis of data from major dairy breeds in Denmark-Finland-Sweden and France.

^2^54K: SNPs in 54K chip.

^3^G1: one-component GBLUP model; B1: one-component Bayesian four-distribution mixture model.

^4^DK: Danish bull as the reference population; DKUS: Danish and US bull as the reference population; COW = Danish cows as the reference population; DKCOW = Danish bull and cows as the reference population. DKUSCOW= Danish and US bulls and Danish cows as the reference population.

^5^Bulls were used as the validation population.
